# Changing trends in the disease burden of non-melanoma skin cancer globally from 1990 to 2019 and its predicted level in 25 years

**DOI:** 10.1186/s12885-022-09940-3

**Published:** 2022-07-30

**Authors:** Wan Hu, Lanlan Fang, Ruyu Ni, Hengchuan Zhang, Guixia Pan

**Affiliations:** Department of Epidemiology and Biostatistics, School of Public Health, Anhui Medical University, 81 Meishan Road, Hefei, 230032 Anhui China

**Keywords:** Non-melanoma skin cancer, Global, Burden, Predict, Trend

## Abstract

**Background:**

The disease burden of non-melanoma skin cancer (NMSC) has become a significant public health threat. We aimed to conduct a comprehensive analysis to mitigate the health hazards of NMSC.

**Methods:**

This study had three objectives. First, we reported the NMSC-related disease burden globally and for different subgroups (sex, socio-demographic index (SDI), etiology, and countries) in 2019. Second, we examined the temporal trend of the disease burden from 1990 to 2019. Finally, we used the Bayesian age-period-cohort (BAPC) model integrated nested Laplacian approximation to predict the disease burden in the coming 25 years. The Norpred age-period-cohort (APC) model and the Autoregressive Integrated Moving Average (ARIMA) model were used for sensitivity analysis.

**Results:**

The disease burden was significantly higher in males than in females in 2019. The results showed significant differences in disease burden in different SDI regions. The better the socio-economic development, the heavier the disease burden of NMSC. The number of new cases and the ASIR of basal cell carcinoma (BCC) were higher than that of squamous cell carcinoma (SCC) in 2019 globally. However, the number of DALYs and the age-standardized DALYs rate were the opposite. There were statistically significant differences among different countries. The age-standardized incidence rate (ASIR) of NMSC increased from 54.08/100,000 (95% uncertainty interval (UI): 46.97, 62.08) in 1990 to 79.10/100,000 (95% UI: 72.29, 86.63) in 2019, with an estimated annual percentage change (EAPC) of 1.78. Other indicators (the number of new cases, the number of deaths, the number of disability-adjusted life years (DALYs), the age-standardized mortality rate (ASMR), and the age-standardized DALYs rate) showed the same trend. Our predictions suggested that the number of new cases, deaths, and DALYs attributable to NMSC would increase by at least 1.5 times from 2020 to 2044.

**Conclusions:**

The disease burden attributable to NMSC will continue to increase or remain stable at high levels. Therefore, relevant policies should be developed to manage NMSC, and measures should be taken to target risk factors and high-risk groups.

**Supplementary Information:**

The online version contains supplementary material available at 10.1186/s12885-022-09940-3.

## Capsule summary

- This is the first systematic assessment and prediction of the disease burden of non-melanoma skin cancer worldwide. The Bayesian age-period-cohort model integrated nested Laplacian approximation is used to predict the disease burden.
- The disease burden will continue to increase or remain relatively stable at high levels in the future.

## Introduction

Skin cancer is a malignant tumor of the skin, and it has become a prominent public health threat [1]. It could be divided into fatal malignant melanoma and less deadly non-melanoma [2]. Non-melanoma skin cancer (NMSC) is the most common type, representing about 1/3 of all malignancies diagnosed worldwide yearly [3]. NMSC is the most common malignancy in people with fair skin, including basal cell carcinoma (BCC) and squamous cell carcinoma (SCC) [4]. It is not suitable for surgical treatment, adjunctive care, or palliative care. It is usually treated with radiotherapy [5, 6]. With an aging population, the incidence attributable to NMSC is increasing [7, 8]. It was estimated that the national health service spent 180 million pounds in 2020 [3]. The average annual cost of treating melanoma in the United States was estimated at $3.3 billion from 2007 to 2011, and the average annual cost of treating NMSC was estimated at $4.8 billion, for a total of $8.1 billion [9]. Current research shows that prevention helps reduce the disease burden, so prevention efforts are positive from every perspective [10]. Therefore, it is urgent to understand the global trend of NMSC. The purpose is to help formulate relevant health policies and guide the practice, prevention, and management of NMSC.

Currently, studies on NMSC mainly focus on clinical treatment [5, 11, 12], and few studies measures the disease burden of NMSC. Aggarwal assessed the burden of skin cancer in the United States from 1990 to 2019. The results showed an increase in the incidence and prevalence of melanoma, BCC, and SCC [9]. Pondicherry reported the incidence of NMSC in the Auckland region of New Zealand [13]. Cakir presented incidence and health care costs for NMSC from Australia, the United States, and Europe. Additionally, he noted that NMSC ranked fifth in cost of care after prostate, lung, colon, and breast cancers [14]. However, these studies were only limited to some countries, and no studies had comprehensively assessed the global disease burden caused by NMSC. When population health measurements are more complex, it is essential to provide a comprehensive assessment of health losses caused by NMSC, with detailed analysis by sex, Global Burden of Disease (GBD) region, economic level, and types.

Therefore, this study aimed to assess the global disease burden of NMSC and predict the global disease burden in the future. The objective was to provide an evidence-based assessment of the effectiveness of current prevention and treatment strategies, make recommendations for future prevention and control policies, and reduce the disease burden of NMSC.

## Methods

### Data sources

The data on deaths, disability-adjusted life years (DALYs), and incidence of NMSC from 1990 to 2019 were extracted from the GBD Study 2019 website (http://www.globalburden.org/). This was free of charge provided by the Institute for Health Metrics and Evaluation (IHME) [15–17]. The GBD study 2019 was a systematic survey that assesses the health effects of diseases, injuries, and risk factors based on age, sex, and GBD region [18]. The details of the methodology had been described in previous publications [19–21]. The following was a brief introduction to the GBD study 2019. First, the period of this study was from 1990 to 2019. Second, the scope of the study was global. All countries and territories were divided into seven super-regions and 21 regions based on geographic contiguity and epidemiological homogeneity. At the same time, all countries and territories were divided into five areas according to the socio-demographic index (SDI) indicator. They were high SDI, high-middle SDI, middle SDI, low-middle SDI, and low SDI. The SDI indicator was a comprehensive measure of developmental level based on average education level, total fertility, and per capita income, ranging from worst zero to best one hundred.

The data of the age-period-cohort (APC) model were as follows. The population forecast data came from the 2019 revised edition of the population of the world outlook (https://population.un.org/wpp/Download/Standard/CSV/). The standardization of the World Health Organization (WHO) in 2000–2025 demographic data came from a public website (https://seer.cancer.gov/stdpopulations/world.who.html/).

### Statistical analysis

This study was a secondary analysis of GBD research results and had three objectives. First, we assessed the NMSC-related disease burden in 2019 and analyzed it by subgroups, including sex, SDI, etiology, and countries. We described the NMSC-related disease burden by using the number and the age-standardized rates of incidence, death, and DALYs. Nonparametric rank-sum tests, including the Mann-Whitney U test and the Kruskal Wallis test, were used to analyze differences in disease burden among subgroups [22]. The significance level was set at 0.05 [22]. Second, we evaluated the trend for disease burden from 1990 to 2019. To reflect the trend of NMSC burden, we used linear regression analysis to calculate the estimated annual percentage change (EAPC) of the age-standardized rates globally and in all subgroups, including sex, SDI, etiology, and GBD regions. The age-standardized rates were based on the GBD reference population. In addition, 21 GBD regions were divided into four categories (a: significant growth; b: a slight increase; c: basically stable or decrease slightly; d: significantly decreased) through cluster analysis [23] to compare the disease burden of NMSC in different GBD regions. Finally, we used the Bayesian age-period-cohort (BAPC) model integrated nested Laplace approximations [24] to predict the disease burden from 2019 to 2044. We applied the Norpred APC model [25] and the Autoregressive Integrated Moving Average (ARIMA) model [26] for sensitivity analysis to verify the stability of the prediction.

All data collation and analysis were performed by R (version 4.0.2) software.

## Results

### Global disease burden assessment for NMSC in 2019

The number of new cases attributable to NMSC was 6,353,687 (95% uncertainty interval (UI): 5,805,441, 6,952,145) in 2019, the number of deaths was 56,054 (95% UI: 50,415, 59,792), and the number of DALYs was 1,183,233 (95% UI: 1,085,365, 1,264,545). The ASIR was 79.10/100,000 (95% UI: 72.29, 86.63), the ASMR was 0.73/100,000 (95% UI: 0.65, 0.78), and the age-standardized DALYs rate was 14.67/100,000 (95% UI: 13.45, 15.67) (**Supplementary Table** **1****–****3**).

**Fig. 1:**
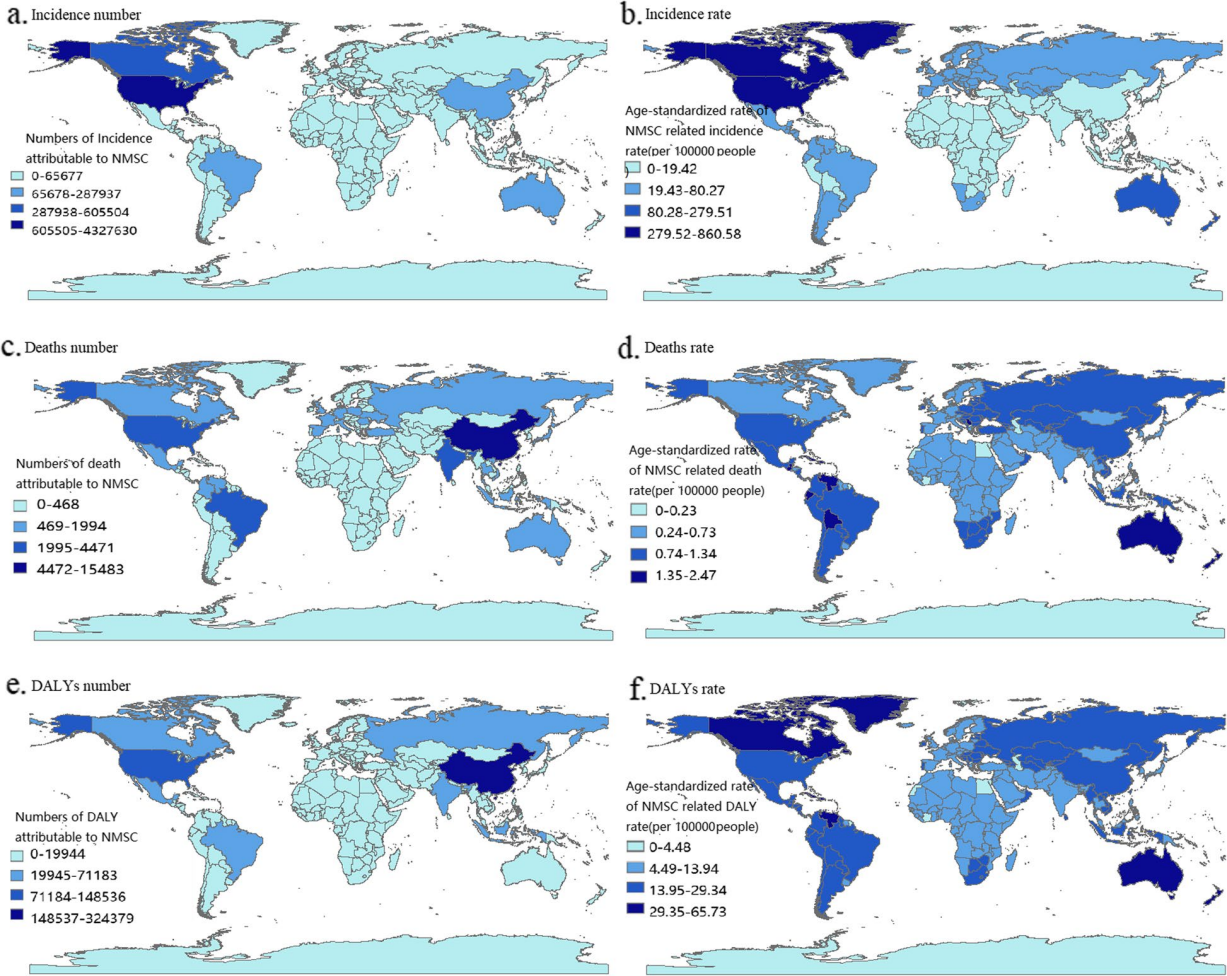
Numbers and age-standardized rates of NMSC-related incidence (**a** and **b**), deaths (**c** and **d**), and DALYs (**e** and **f**) across countries. Abbreviations: DALYs: disability-adjusted life years; NMSC: non-melanoma skin cancer

The disease burden was significantly higher in males than in females in 2019. The results of the Kruskal Wallis test showed significant differences in disease burden among different SDI regions (**Supplementary Table** **1****–****3**). We found that regions with better socioeconomic development had a greater disease burden from NMSC in 2019. As for the subgroup analysis of etiology, the number of new cases and the ASIR of BCC were higher than that of SCC in 2019 globally. The number of DALYs and the age-standardized DALYs rate of BCC were lower than SCC in 2019. We could not compare the number of deaths and the ASMR between the two etiologies due to the lack of data for BCC in the database. The global disease burden for different countries in 2019 is shown in Fig. 1. We found that the United States had the highest number of new cases attributable to NMSC. The ASIR was high in North America except in Mexico. China had the highest number of deaths and DALYs of all countries and territories. The ASMR was severe in Australia and a few countries in South America. Furthermore, the age-standardized DALYs rate was highest in Canadian, the Arctic, and Australia.

### Global disease burden assessment for NMSC from 1990 to 2019

The number of new cases attributable to NMSC increased from 1,951,299 (95% UI: 1,692,794, 2,237,075) in 1990 to 6,353,687 (95% UI: 5,805,441, 6,952,145) in 2019, and the number of deaths of NMSC increased from 23,222 (95% UI: 21,441, 24,436) to 56,054 (95% UI: 50,415, 59,792) between 1990 and 2019. The number of DALYs also exhibited a upward trend, which increase significantly from 561,854 (95% UI: 518,874, 599,141) in 1990 to 1,183,233 (95% UI: 1,085,365, 1,264,545) in 2019. The age-standardized incidence rate (ASIR) of NMSC increased from 54.08/100,000 (95% UI: 46.97, 62.08) in 1990 to 79.10/100,000 (95% UI: 72.29, 86.63) in 2019, with an EAPC of 1.78 (95% confidence interval (CI): 1.35, 2.21). The age-standardized mortality rate (ASMR) of NMSC increased significantly from 0.69/100,000 (95% UI: 0.63, 0.73) to 0.73/100,000 (95% UI: 0.65, 0.78) between 1990 and 2019, with an EAPC of 0.41 (95% CI: 0.34 to 0.49), and the age-standardized DALYs rate significantly increased from 14.44/100,000 (95% UI: 13.31, 15.42) to 14.67/100,000 (95% UI: 13.45, 15.67) during the same period, with an EAPC of 0.20 (95% CI: 0.10 to 0.30) (**Supplementary Table** **1****–****3**).

In subgroup analyses, the EAPC values of the age-standardized rates had significant differences between the genders from 1990 to 2019. The increase in disease burden was more significant for males than females (**Supplementary Table** **1****–****3**). From 1990 to 2019, the number of new cases, deaths, and DALYs attributable to NMSC increased in all regions regardless of the level of SDI. The ASIR of NMSC also increased in the five SDI regions (high SDI, high-middle SDI, middle SDI, low-middle SDI, and low SDI). However, the ASMR decreased in the high-middle SDI region and remained stable in the high SDI region from 1990 to 2019. The age-standardized DALYs rate decreased in the high-middle SDI region. There were significant differences in NMSC-related disease burden between the etiologies. Over time, all disease burden indicators (the number of new cases, the ASIR, the number of deaths, the ASMR, the number of DALYs, and the age-standardized DALYs rate) of SCC showed an upward trend from 1990 to 2019. Furthermore, some of the disease burden indicators (the number of new cases, the ASIR, the number of DALYs, and the age-standardized DALYs rate) of BCC showed an upward trend (**Supplementary Table** **1****–****3**). Regarding geographical GBD regions, the disease burden of NMSC varied significantly between GBD regions (**Supplementary Table** **1****–****3**). The results of cluster analysis are shown in **Supplementary Fig.** **1**. The regions which had significant growth in EAPC of AMIR from 1990 to 2019 were East Asia and High-income North America. The Caribbean and Southeast Asia were in the group of significantly decreased EAPC values. The EAPC of AMDR increased most in Central Asia and decreased most in Central Europe. As for the age-standardized DALYs rate, the region which had significant growth in EAPC of AMIR from 1990 to 2019 was Central Asia. Central Europe and High-income Asia Pacific were in the group of significantly decreased EAPC values.

### Global disease burden prediction for NMSC

**Fig. 2:**
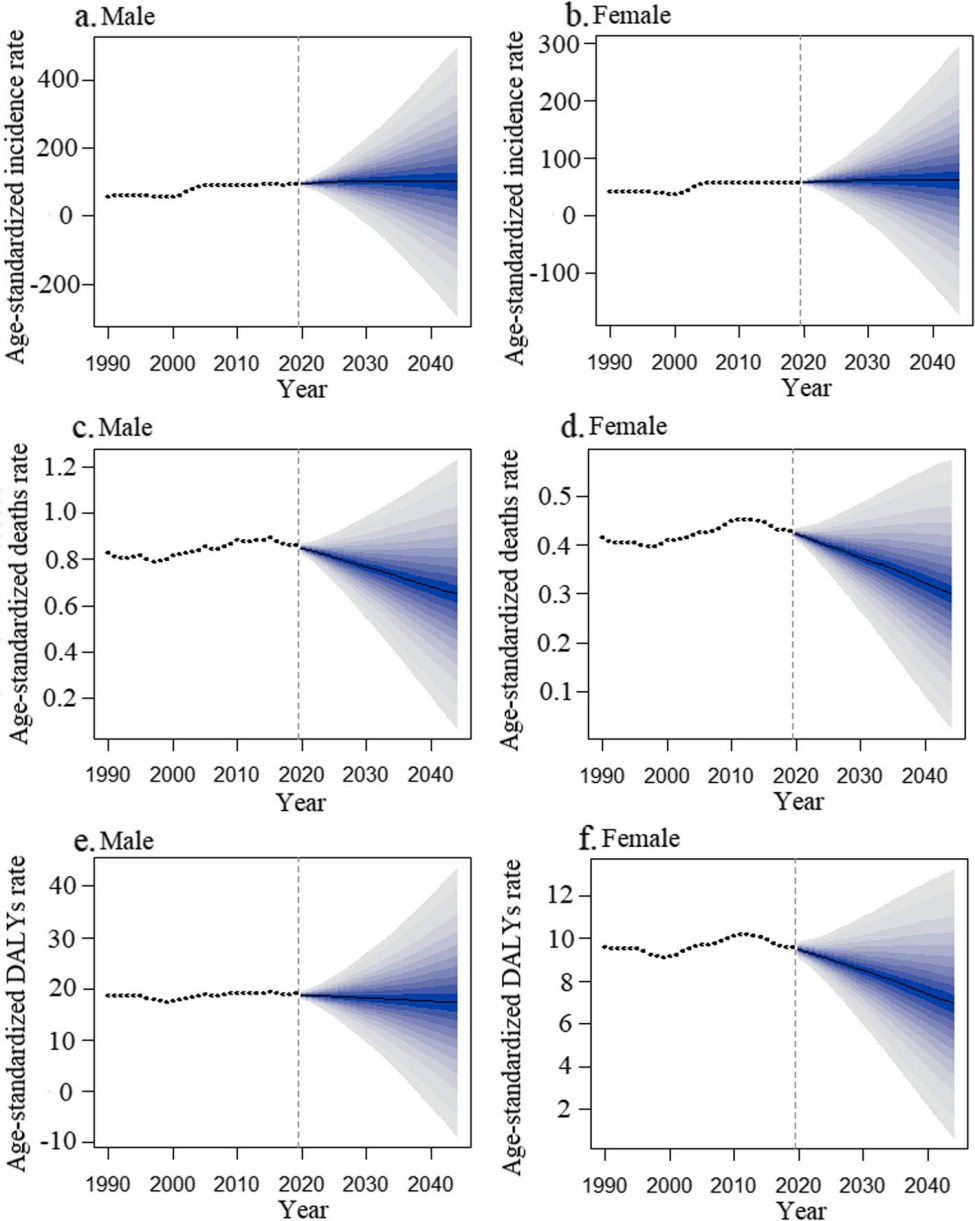
Trends in the NMSC-related ASIR **(a** and **b)**, ASMR **(c** and **d)**, and the age-standardized DALYs **(e** and **f)** by sex globally: observed (dashed lines) and predicted rates of the BAPC model (solid lines). The blue region shows the upper and lower limits of the 95% UIs. **Abbreviations:** DALYs: disability-adjusted life years; NMSC: non-melanoma skin cancer; BAPC: Bayesian age-period-cohort; UIs: uncertainty intervals

**Fig. 3:**
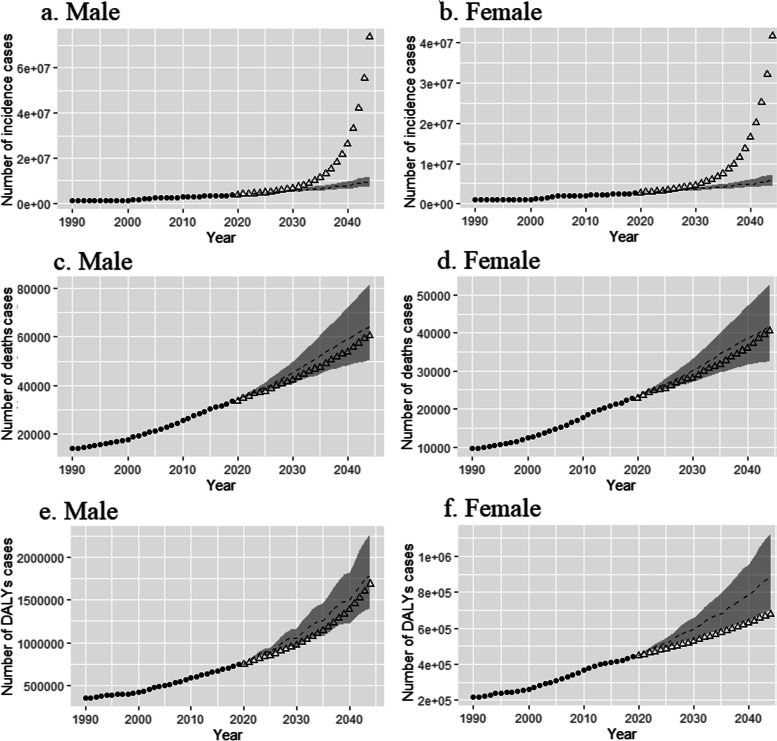
Trends in NMSC-related numbers of incidence cases **(a** and **b)**, deaths cases **(c** and **d)**, and DALYs cases **(e** and **f)** by sex globally: observed (before 2019) and predicted (after 2019) numbers. Shading indicates if the rate remained stable (baseline reference), decreased by 1% per year (optimistic reference, lower limit), and increased by 1% per year (pessimistic reference, upper limit) based on the observed rate in 2019. The curve formed by the triangle is the prediction result of the BAPC model. **Abbreviations:** DALYs: disability-adjusted life years; NMSC: non-melanoma skin cancer; BAPC: Bayesian age-period-cohort

**Fig. 4:**
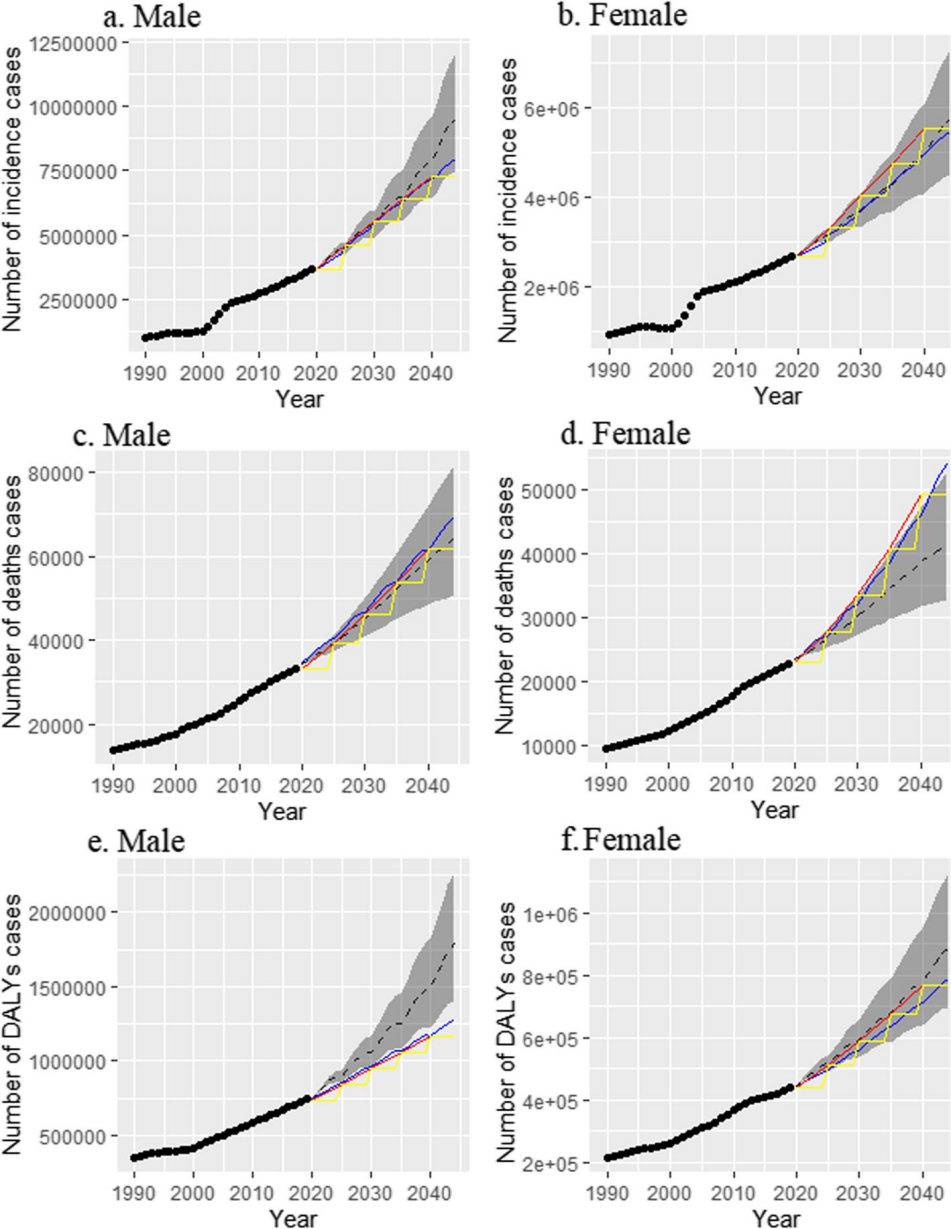
Trends in NMSC-related number of incidence cases **(a** and **b)**, deaths cases **(c** and **d)**, and DALYs cases **(e** and **f)** by sex globally: observed (before 2019) and predicted (after 2019) numbers. Shading indicates if the rate remained stable (baseline reference), decreased by 1% per year (optimistic reference, lower limit), and increased by 1% per year (pessimistic reference, upper limit) based on the observed rate in 2019. Three methods are used in the prediction. The red line is calculated by the predicted rate of each 5 years group and the average population size of the 5 year group. The blue line method is to calculate the rate of each group in terms of the predicted rate of each 5 years group and the average population situation of the 5 year group. The yellow line is calculated by the predicted rate of each 5 years group and the annual population situation. **Abbreviations:** DALYs: disability-adjusted life years; NMSC: non-melanoma skin cancer

**Fig. 5:**
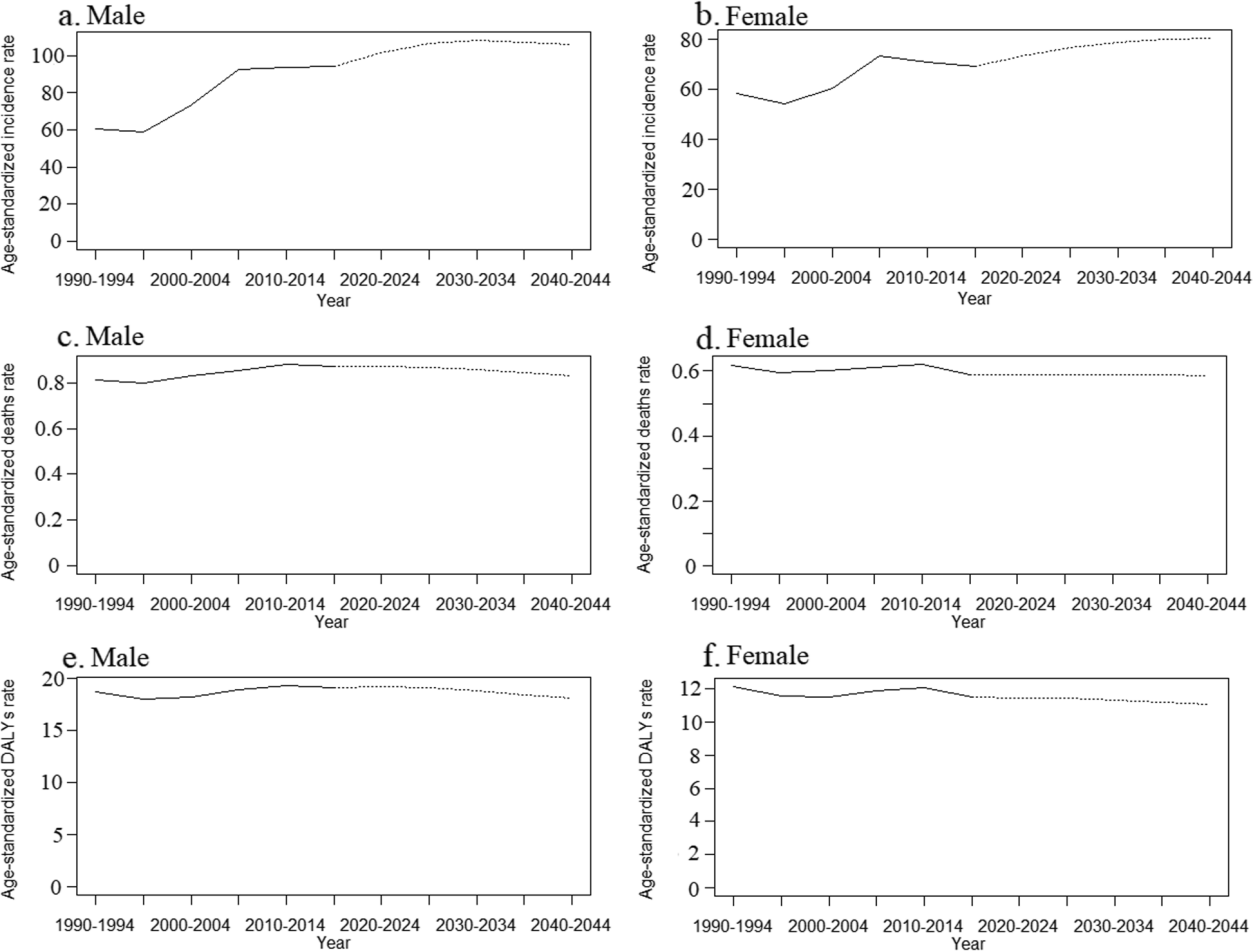
Trends in NMSC-related incidence **(a** and **b)**, deaths **(c** and **d)**, and DALYs rates **(e** and **f)** by sex globally: observed (solid lines) and predicted rates of the Norpred APC model (dashed lines). **Abbreviations:** DALYs: disability-adjusted life years; NMSC: non-melanoma skin cancer; APC: age-period-cohort

The BAPC model predicts that the ASIR attributable to NMSC will increase slightly for both sexes over the next 25 years, but the ASMR and the age-standardized DALYs rate will decrease (Fig. 2**and Supplementary Table** **4**). The number of new cases, deaths, and DALYs will increase over the next 25 years due to population growth and aging. The shadows in the figures show that if the corresponding rates increase or decrease by 1% per year, the number of new cases and deaths could change dramatically. This further highlights the importance of NMSC prevention and treatment. The results show that the number of new cases of NMSC for males will increase from 3,682,933 in 2019 to 73,642,458 in 2044, an approximately 20-fold increase. The number of deaths will increase from 33,244 to 60,575 between 2019 and 2044, an increase of about 1.82 times. The number of DALYs will increase from 742,528 to 1,688,448 during the same period, an increase of 2.27 times. The number of new cases among females will increase approximately 15.57-fold, from 2,670,753 in 2019 to 41,587,505 in 2044. The number of deaths will increase from 22,809 to 40,550, an increase of about 1.78 times. The number of DALYs will increase from 440,704 to 680,152, approximately 1.54 times (Fig. 3**and Supplementary Table** **4**). The results of the Norpred APC model are consistent with the above results. The results show that the ASIR of both genders shows an uptrend and the age-standardized DALYs rate shows a downtrend in the next 25 years. The ASMR of males shows a decreasing trend, while the ASMR of females remains unchanged (Fig. 4-5). As shown in the figure, the predicted results of the ARIMA model show that the age-standardized rate remains relatively stable. This is different from the prediction of the BAPC model. However, in terms of the quantitative burden of disease indicators, the number of new cases and deaths for both males and females will increase over the next 25 years. This is consistent with the prediction of the APC model. The inconsistent results may be because the data is summarized yearly and is somewhat crude and sparse (**Supplementary Fig.** **2****and Supplementary Fig.** **3**).

## Discussion

This study comprehensively assesses and predicts the global NMSC-related disease burden and makes notable discoveries. Globally, the disease burden of NMSC is severe, underscoring that NMSC is a severe threat to global human health. Our results are consistent with some other studies [27, 28]. Ferlay shows that worldwide estimated mortality rates for all types of NMSC were higher than the corresponding mortality rates for melanoma, mesothelioma, oropharyngeal, and thyroid cancers [29]. NMSC causes a substantial economic burden worldwide [30, 31]. It was the most expensive cancer in Australia, with an expenditure of $511 million in 2010 [32]. In the United States, it is estimated that the total annual spending related to NMSC was 650 million US dollars, and the cost of health insurance is 6–7 times that of treating melanoma [9]. Furthermore, the disease burden has increased in recent years [32, 33]. Between 1997 and 2010, the treatment of NMSC increased by 86% [34]. Madan reports that NMSC incidence and death rates are rising [35]. The incidence of BCC shows a continuous linear increase [36]. This phenomenon might be related to several factors. First, our society presents a trend of an aging population, and the elderly are a high-risk group for NMSC [37]. Additionally, exposure to UV radiation at work and play has a significant impact [38–40]. In females younger than 25 years, activity-induced tanning is associated with a significantly increased risk of BCC and SCC in this population [41]. However, the current research focuses on some traditional cancers, so a full assessment of the global burden of NMSC is necessary.

Sex differences in the onset of many diseases have been documented, such as diabetic cardiomyopathy [42], multiple scleroses [43], and myeloid leukemia [44]. There also appears to be gender differences in disease burden attributable to NMSC. Previous studies and the latest results from the National Cancer Institute show that cancer incidence and death rates are higher in males than females in all GBD regions [45]. Epidemiological studies have reported that the disease burden of NMSC is significantly higher in males than in females [46–49]. According to the American Cancer Society, NMSC is twice as common in males as in females, with SCC three times more common in males than females [50]. A population-based study assessing trends in NMSC mortality shows that males were twice as likely to die as females in the United States [9]. This might be because males and females have different ways of working and living. Males tend to work outdoors, exposing them to more ultraviolet rays. Males are also less likely than females to use sunscreen, hats, and other protective gear. Occupational ultraviolet exposure is strongly associated with the disease burden of NMSC [51].

The risk of NMSC varies with social indicators, work environment, occupational class, and education level [52–54]. In a national study of NMSC incidence and survival, results show that higher socioeconomic status is strongly associated with a higher risk of BCC in the population. The higher the socioeconomic status, the higher the risk of BCC [55]. This is consistent with the results of this study. This might be because tanning is more and more favored by society and regarded as a symbol of happiness and success with the development of community and economy. In addition, people have more leisure time to do outdoor activities and even go to the seaside for holidays. Another possible explanation is that in areas with a higher SDI, people know more about NMSC and are more likely to be checked out. This leads to more detection and reporting.

Since NMSC is only categorized as two types of BCC and SCC in the GBD study 2019, we assess the disease burden caused by these two types. Other studies show that BCC has the highest incidence but is rarely fatal [29, 56], which is consistent with our research. This might be because that SCC is primarily associated with total and occupational sun exposure. In contrast, BCC is related to non-occupational or recreational sun exposure [1].

We find that the NMSC-related disease burden varies in different GBD regions. NMSC appears to be directly related to skin types in Caucasians [57]. NMSC is the most common malignancy in people with fair skin [58]. Residents are at significantly higher risk of skin cancer in some areas with high solar exposure [19]. Studies have shown a higher disease burden in European descent, such as in Australia, New Zealand, North America, and Northern Europe [29]. Therefore, it is necessary for us to focus more on these high-risk areas and conduct more health education campaigns to control and reduce the disease burden of NMSC.

In this study, we present that the number of new cases, prevalence, deaths, and DALYs will continue to increase. This is likely the combined result of increasing high-risk behaviors (including outdoor recreation) and changing demographics over the following years. Studies show that NMSC increases by 2–3% annually in the United States [59]. Globally, the incidence rate of BCC has been growing and is predicted to continue to grow until at least 2040 [16]. It is speculated that this might be due to more exposure to the outdoors for recreational and social reasons. In addition, Mushtaq points out that the incidence of NMSC is increasing, which is consistent with our study [60]. He also points out that this is attributed to the increased use of sunbeds, recreational sun exposure, and the aging population. Although the current measures and strategies of new medical management have achieved specific results. But overall, the disease burden remains severe. Furthermore, due to the aging trend of the population, high priority should be given to NMSC.

However, this study has some limitations. First, the assessment of the disease burden is carried out at the country and GBD region levels. However, some countries are vast, and the burden of disease could vary significantly between different provinces in a country. Second, the GBD database has defects such as data quality assurance. Finally, due to the lack of data for BCC in the database, we could not compare the number of deaths and the ASMR between the two etiologies. Therefore, we will further assess the trend of the disease burden in different GBD regions of countries. Beyond that, it is necessary for us to better translate our research into action and develop public policies.

## Conclusion

This study shows that NMSC poses a substantial global disease burden and predicts that the future disease burden of NMSC will remain severe. We call on health policymakers to act and intervene. They also could develop more targeted and effective policies and measures to reduce adverse health effects associated with NMSC. These policies include enhancing the management and prevention of NMSC-related risk factors and focusing on high-risk groups.

## Supplementary Information

**Additional file 1 Supplementary Table 1.** The number of new cases and the ASIR of NMSC in 1990 and 2019, and its temporal trends from 1990 to 2019. Abbreviations: NMSC, non-melanoma skin cancer; ASIR, age-standardized incidence rate. **Supplementary Table 2.** The number of deaths and the ASMR of NMSC in 1990 and 2019, and its temporal trends from 1990 to 2019. Abbreviations: NMSC, non-melanoma skin cancer; ASMR, age-standardized mortality rate. **Supplementary Table 3.** The number of DALYs rate and the age-standardized DALYs rate of NMSC in 1990 and 2019, and its temporal trends from 1990 to 2019. Abbreviations: NMSC, non-melanoma skin cancer; DALYs, disability-adjusted life years. **Supplementary Table 4.** Global trends in the number of new cases, the number of deaths, the number of DALYs, the ASIR, the ASMR, and the age-standardized DALYs rate by sex from 2019 to 2044 predicted by the BAPC model. **Abbreviations:** ASIR, age-standardized incidence rate; ASMR, age-standardized mortality rate; DALYs, disability-adjusted life years; BAPC, Bayesian age-period-cohort.

**Additional file 2 Supplementary Fig. 1.** Results of cluster analysis (a: significant growth; b: a slight increase; c: basically stable or decrease slightly; d: significantly decreased) based on the EAPC values of the ASIR **(A)**, the ASMR **(B)**, and the age-standard DALYs rate **(C)** from 1990 to 2019. **Abbreviations:** EAPC, estimated annual percentage change; ASIR, age-standardized incidence rate; ASMR, age-standardized mortality rate; DALY, disability-adjusted-life-year.

**Additional file 3 Supplementary Fig. 2.** Trends in the number of new cases **(a and b)**, the number of deaths **(c and d)**, and the number of DALYs **(e and f)** by genders globally: observed (before 2019) and predicted numbers of the ARIMA model (after 2019). Shading indicates the upper and lower limits of the 95% CIs. **Abbreviations:** DALYs, disability-adjusted-life-years; CIs, confidence intervals.

**Additional file 4 Supplementary Fig. 3.** Trends in the ASIR **(a and b)**, the ASMR **(c and d)**, and the age-standardized DALYs rate **(e and f)** by genders globally: observed (before 2019) and predicted rates of the ARIMA model (after 2019). Shading indicates the upper and lower limits of the 95% CIs. **Abbreviations:** ASIR, age-standardized incidence rate; ASMR, age-standardized mortality rate; DALYs, disability-adjusted-life-years; CIs, confidence intervals.

## Data Availability

The data underlying this article will be shared on reasonable request to the corresponding author. The data on deaths, DALYs, and incidence of NMSC from 1990 to 2019 are extracted from the GBD Study 2019 website (http://www.globalburden.org/). The population forecast data come from the 2019 revised edition of the population of the world outlook (https://population.un.org/wpp/Download/Standard/CSV/). The standardization of the World Health Organization (WHO) in 2000–2025 demographic data is from a public website (https://seer.cancer.gov/stdpopulations/world.who.html/).

## Abbreviations

NMSC: Non-melanoma skin cancer
BCC: Basal cell carcinoma
SCC: Squamous cell carcinoma
DALYs: Disability-adjusted life years
WHO: World Health Organization
BAPC: Bayesian age-period-cohort
APC: Age-period-cohort
GBD: Global Burden of Disease
EAPC: Estimated annual percentage change
ASIR: Age-standardized incidence rate
ASMR: Age-standardized mortality rate
IHME: The Institute for Health Metrics and Evaluation
ARIMA: Autoregressive Integrated Moving Average
UI: Uncertainty interval
CI: Confidence interval
SDI: Socio-demographic index
