# Supplementary material for: Changing trends in the disease burden of non-melanoma skin cancer globally from 1990 to 2019 and its predicted level in 25 years
Source: BMC Cancer. 2022 Jul 30;22:836. doi: 10.1186/s12885-022-09940-3 (PMC9339183; doi:10.1186/s12885-022-09940-3)
Supplement: Supplementary file 1 — Additional file 1 Supplementary Table 1. The number of new cases and the ASIR of NMSC in 1990 and 2019, and its temporal trends from 1990 to 2019. Abbreviations: NMSC, non-melanoma skin cancer; ASIR, age-standardized incidence rate. Supplementary Table 2. The number of deaths and the ASMR of NMSC in 1990 and 2019, and its temporal trends from 1990 to 2019. Abbreviations: NMSC, non-melanoma skin cancer; ASMR, age-standardized mortality rate. Supplementary Table 3. The number of DALYs rate and the age-standardized DALYs rate of NMSC in 1990 and 2019, and its temporal trends from 1990 to 2019. Abbreviations: NMSC, non-melanoma skin cancer; DALYs, disability-adjusted life years. Supplementary Table 4. Global trends in the number of new cases, the number of deaths, the number of DALYs, the ASIR, the ASMR, and the age-standardized DALYs rate by sex from 2019 to 2044 predicted by the BAPC model. Abbreviations: ASIR, age-standardized incidence rate; ASMR, age-standardized mortality rate; DALYs, disability-adjusted life years; BAPC, Bayesian age-period-cohort. [file 12885_2022_9940_MOESM1_ESM.docx]

**Changing trends in the disease burden of non-melanoma skin cancer globally from 1990 to 2019 and its predicted level in 25 years**

Wan Hu^a^, Lanlan Fang^a^, Ruyu Ni^a^, Hengchuan Zhang^a^, Guixia Pan^a,*^

^a^ Department of Epidemiology and Biostatistics, School of Public Health, Anhui Medical University, 81 Meishan Road, Hefei, Anhui 230032, China.

^*^ Corresponding author at: Department of Epidemiology and Biostatistics, School of Public Health.

**Corresponding author:**

Full name: Guixia Pan

Postal address: Anhui Medical University, 81 Meishan Road, Hefei, Anhui 230032, China.

E-mail address: pgxkd@163.com

**Figure legends**

**Supplementary Figure 1.** Results of cluster analysis (a: significant growth; b: a slight increase; c: basically stable or decrease slightly; d: significantly decreased) based on the EAPC values of the ASIR **(A)**, the ASMR **(B)**, and the age-standard DALYs rate **(C)** from 1990 to 2019. **Abbreviations:** EAPC, estimated annual percentage change; ASIR, age-standardized incidence rate; ASMR, age-standardized mortality rate; DALY, disability-adjusted-life-year.

**Supplementary Figure 2.** Trends in the number of new cases **(a and b)**, the number of deaths **(c and d)**, and the number of DALYs **(e and f)** by genders globally: observed (before 2019) and predicted numbers of the ARIMA model (after 2019). Shading indicates the upper and lower limits of the 95% CIs. **Abbreviations:** DALYs, disability-adjusted-life-years; CIs, confidence intervals.

**Supplementary Figure 3.** Trends in the ASIR **(a and b)**, the ASMR **(c and d)**, and the age-standardized DALYs rate **(e and f)** by genders globally: observed (before 2019) and predicted rates of the ARIMA model (after 2019). Shading indicates the upper and lower limits of the 95% CIs. **Abbreviations:** ASIR, age-standardized incidence rate; ASMR, age-standardized mortality rate; DALYs, disability-adjusted-life-years; CIs, confidence intervals.

**Table legends**

**Supplementary Table 1.** The number of new cases and the ASIR of NMSC in 1990 and 2019, and its temporal trends from 1990 to 2019. Abbreviations: NMSC, non-melanoma skin cancer; ASIR, age-standardized incidence rate.

**Supplementary Table 2.** The number of deaths and the ASMR of NMSC in 1990 and 2019, and its temporal trends from 1990 to 2019. Abbreviations: NMSC, non-melanoma skin cancer; ASMR, age-standardized mortality rate.

**Supplementary Table 3.** The number of DALYs rate and the age-standardized DALYs rate of NMSC in 1990 and 2019, and its temporal trends from 1990 to 2019. Abbreviations: NMSC, non-melanoma skin cancer; DALYs, disability-adjusted life years.

**Supplementary Table 4.** Global trends in the number of new cases, the number of deaths, the number of DALYs, the ASIR, the ASMR, and the age-standardized DALYs rate by sex from 2019 to 2044 predicted by the BAPC model. **Abbreviations:** ASIR, age-standardized incidence rate; ASMR, age-standardized mortality rate; DALYs, disability-adjusted life years; BAPC, Bayesian age-period-cohort.

**Supplementary Table 1.** The number of new cases and the ASIR of NMSC in 1990 and 2019, and its temporal trends from 1990 to 2019. **Abbreviations:** NMSC, non-melanoma skin cancer; ASIR, age-standardized incidence rate.

| Characteristics | 1990 | | 2019 | | 1990–2019 | p-value |
| --- | --- | --- | --- | --- | --- | --- |
|  | Number of new cases(95% UI) | ASIR/100000(95% UI) | Number of new cases(95% UI) | ASIR/100000(95% UI) | EAPC(95% CI) |  |
| **Overall** | 1951299(1692794,2237075) | 54.08(46.97,62.08) | 6353687(5805441,6952145) | 79.10(72.29,86.63) | 1.78 (1.35 to 2.21) |  |
| **Sex** |  |  |  |  |  |  |
| Male | 1011240(876569,1171170) | 65.49(56.95,75.87) | 3682933(3345235,4055580) | 102.81(93.87,112.91) | 2.07 (1.62 to 2.53) | <0.001 |
| Female | 940059(815820,1079205) | 46.50(40.60,52.91) | 2670753(2434525,2912469) | 61.15(55.75,66.68) | 1.35 (0.94 to 1.76) |  |
| **Socio-demographic index** |  |  |  |  |  |  |
| Low SDI | 9581(7697,11826) | 3.95(3.22,4.73) | 21710(17624,26774) | 3.98(3.29,4.77) | 0.26 (0.15 to 0.36) | <0.001 |
| Low-middle SDI | 36279(30502,42667) | 6.44(5.47,7.54) | 112256(93483,131901) | 8.33(7.00,9.74) | 0.88 (0.78 to 0.98) |  |
| Middle SDI | 92378(78839,107834) | 9.31(7.96,10.76) | 326774(276655,378560) | 13.33(11.43,15.36) | 1.43 (1.24 to 1.61) |  |
| High-middle SDI | 236726(208971,268701) | 23.09(20.48,25.98) | 529706(459348,606608) | 26.30(22.88,29.97) | 0.47 (0.38 to 0.56) |  |
| High SDI | 1575994(1354868,1822928) | 152.43(131.62,175.59) | 5344905(4893803,5830519) | 288.67(264.66,313.72) | 2.83 (2.31 to 3.35) |  |
| **Etiology** |  |  |  |  |  |  |
| basal-cell carcinoma | 1194817(1003244,1414118) | 31.82(26.70,37.62) | 3951466(3488307,4463602) | 48.80(43.11,55.01) | 2.23 (1.75 to 2.72) | <0.001 |
| squamous-cell carcinoma | 756481(606882,933155) | 22.27(18.04,27.43) | 2402221(2122698,2712803) | 30.30(26.89,34.08) | 1.13 (0.50 to 1.77) |  |
| **Region** |  |  |  |  |  |  |
| Andean Latin America | 3913 (3558,4287) | 19.63(17.88,21.51) | 10160(8389,12068) | 18.28(15.08,21.78) | -0.09 (-0.37 to 0.19) | <0.001 |
| Australasia | 53551(46468,61163) | 232.14(202.22,263.69) | 140176(120430,163163) | 279.19(238.87,324.97) | 0.53 (0.39 to 0.67) |  |
| Caribbean | 2613(2220,3028) | 10.10(8.58,11.70) | 4790(4066,5548) | 9.25(7.87,10.72) | -0.30 (-0.39 to -0.22) |  |
| Central Asia | 11308(9165,13494) | 24.70(20.17,29.32) | 18486(14803,22362) | 25.73(20.94,30.67) | 0.16 (0.14 to 0.18) |  |
| Central Europe | 38542(34551,43089) | 26.96(24.25,29.96) | 72017(61575,83186) | 33.97(29.26,38.91) | 0.97 (0.84 to 1.10) |  |
| Central Latin America | 28357(23861,33406) | 34.04(28.59,39.78) | 80375(66900,94260) | 34.23(28.62,40.28) | 0.06 (0.04 to 0.07) |  |
| Central Sub-Saharan Africa | 1232(958,1547) | 5.34(4.26,6.43) | 3013(2354,3790) | 5.35(4.30,6.45) | 0.03 (0.00 to 0.06) |  |
| East Asia | 40175 (33832,48102) | 4.58(3.89,5.36) | 288654(247475,329428) | 13.86(11.96,15.79) | 3.81 (3.34 to 4.29) |  |
| Eastern Europe | 50291(41429,59579) | 18.57(15.45,21.78) | 75345(62153,90000) | 22.14(18.25,26.31) | 0.59 (0.55 to 0.64) |  |
| Eastern Sub-Saharan Africa | 3599(2899,4448) | 4.63(3.79,5.53) | 8081 (6517,10031) | 4.64(3.79,5.52) | 0.00 (-0.01 to 0.01) |  |
| High-income Asia Pacific | 6661(5676,7807) | 3.51(3.03,4.09) | 24288(21235,28080) | 5.16(4.49,5.94) | 1.01 (0.89 to 1.13) |  |
| High-income North America | 1354511(1149626,1589401) | 385.57(329.94,448.42) | 4933624(4513848,5371060) | 795.71(729.51,863.95) | 3.23 (2.61 to 3.85) |  |
| North Africa and Middle East | 14262(12110,16511) | 8.46(7.25,9.71) | 34274(28334,40526) | 7.99(6.68,9.40) | 0.04 (-0.23 to 0.31) |  |
| Oceania | 20(16,25) | 0.79(0.70,0.89) | 51(42,62) | 0.83(0.74,0.93) | 0.20 (0.18 to 0.23) |  |
| South Asia | 10039(7854,13115) | 1.67(1.35,2.06) | 25793(20824,32174) | 1.80(1.47,2.17) | 0.27 (0.23 to 0.32) |  |
| Southeast Asia | 7700(6780,8755) | 3.13(2.79,3.50) | 14698(12125,17614) | 2.51(2.12,2.94) | -0.45 (-0.67 to -0.24) |  |
| Southern Latin America | 13052(11588,14601) | 28.95(25.80,32.30) | 26980(23092,31214) | 32.47(27.75,37.35) | 0.24 (0.08 to 0.40) |  |
| Southern Sub-Saharan Africa | 5923(4923,7065) | 20.93(17.46,24.76) | 15216(12694,18071) | 26.36(22.14,31.02) | 0.42 (-0.19 to 1.02) |  |
| Tropical Latin America | 64759(57420,73025) | 70.10(61.99,79.24) | 190659(166747,216057) | 78.47(68.67,88.95) | 0.54 (0.41 to 0.67) |  |
| Western Europe | 237896(214544,264983) | 42.18(38.13,46.52) | 380430(320941,448805) | 43.50(36.37,50.90) | 0.42 (0.26 to 0.59) |  |
| Western Sub-Saharan Africa | 2895(2264,3660) | 3.08(2.44,3.77) | 6576(5131,8400) | 3.15(2.51,3.84) | 0.09 (0.07 to 0.11) |  |

**Supplementary Table 2.** The number of deaths and the ASMR of NMSC in 1990 and 2019, and its temporal trends from 1990 to 2019. **Abbreviations:** NMSC, non-melanoma skin cancer; ASMR, age-standardized mortality rate.

| Characteristics | 1990 | | 2019 | | 1990–2019 | P-value |
| --- | --- | --- | --- | --- | --- | --- |
|  | Number of deaths cases(95% UI) | ASMR/100000 (95% UI) | Number of deaths cases(95% UI) | ASMR/100000 (95% UI) | EAPC(95% CI) |  |
| **Overall** | 23222(21441,24436) | 0.69(0.63,0.73) | 56054(50415,59792) | 0.73(0.65,0.78) | 0.41 (0.34 to 0.49) |  |
| **Sex** |  |  |  |  |  |  |
| Male | 13696(12374,14508) | 0.95(0.86,1.01) | 33244(30347,35571) | 1.01(0.91,1.08) | 0.40 (0.33 to 0.48) | <0.001 |
| Female | 9526(8681,10355) | 0.50(0.45,0.54) | 22809(19350,25231) | 0.52(0.44,0.58) | 0.36 (0.26 to 0.45) |  |
| **Socio-demographic index** |  |  |  |  |  |  |
| Low SDI | 701(466,838) | 0.39(0.24,0.47) | 1968(1308,2347) | 0.48(0.31,0.57) | 0.80 (0.75 to 0.85) | <0.001 |
| Low-middle SDI | 2657(2142,3028) | 0.57(0.45,0.65) | 7865(6864,8605) | 0.69(0.59,0.76) | 0.90 (0.77 to 1.04) |  |
| Middle SDI | 6009(5529,6491) | 0.73(0.66,0.78) | 17710(15838,19361) | 0.84(0.75,0.92) | 0.85 (0.69 to 1.01) |  |
| High-middle SDI | 7641(7187,7973) | 0.87(0.80,0.91) | 15469(13914,16567) | 0.80(0.71,0.86) | -0.22 (-0.28 to -0.16) |  |
| High SDI | 6199(5759,6394) | 0.61(0.56,0.63) | 13002(11102,13900) | 0.61(0.53,0.64) | 0.20 (0.09 to 0.31) |  |
| **Etiology** |  |  |  |  |  |  |
| basal-cell carcinoma |  |  |  |  |  | <0.001 |
| squamous-cell carcinoma | 23222(21441,24436) | 0.69(0.63,0.73) | 56054(50415,59792) | 0.73(0.65,0.78) | 0.41 (0.34 to 0.49) |  |
| **Region** |  |  |  |  |  |  |
| Andean Latin America | 145(128,165) | 0.81(0.71,0.91) | 569(467,677) | 1.06(0.87,1.27) | 1.12 (0.96 to 1.27) | <0.001 |
| Australasia | 324(301,346) | 1.44(1.32,1.54) | 927(804,1011) | 1.70(1.48,1.85) | 0.39 (0.28 to 0.51) |  |
| Caribbean | 269(247,287) | 1.15(1.05,1.23) | 739(623,853) | 1.41(1.19,1.63) | 0.97 (0.84 to 1.09) |  |
| Central Asia | 197(168,216) | 0.47(0.39,0.52) | 476(404,522) | 0.83(0.70,0.91) | 2.72 (2.43 to 3.01) |  |
| Central Europe | 1774(1602,1845) | 1.47(1.30,1.54) | 2366(2056,2657) | 1.08(0.94,1.21) | -1.33 (-1.52 to -1.13) |  |
| Central Latin America | 922(853,961) | 1.28(1.16,1.34) | 2757(2343,3152) | 1.23(1.04,1.40) | -0.23 (-0.28 to -0.18) |  |
| Central Sub-Saharan Africa | 73(47,93) | 0.46(0.27,0.60) | 227(149,298) | 0.57(0.36,0.75) | 0.83 (0.75 to 0.91) |  |
| East Asia | 5180(4578,5805) | 0.70(0.62,0.78) | 15905(13481,18220) | 0.87(0.74,0.99) | 1.39 (1.09 to 1.69) |  |
| Eastern Europe | 1831(1712,1961) | 0.75(0.70,0.81) | 2894(2573,3233) | 0.86(0.76,0.96) | 0.28 (0.03 to 0.53) |  |
| Eastern Sub-Saharan Africa | 266(153,335) | 0.48(0.25,0.62) | 814(450,1029) | 0.66(0.34,0.84) | 1.23 (1.17 to 1.29) |  |
| High-income Asia Pacific | 729(682,760) | 0.43(0.39,0.45) | 2072(1656,2306) | 0.36(0.30,0.40) | -0.57 (-0.65 to -0.49) |  |
| High-income North America | 2528(2335,2609) | 0.72(0.66,0.74) | 5000(4332,5316) | 0.75(0.66,0.79) | 0.29 (0.16 to 0.43) |  |
| North Africa and Middle East | 667(563,778) | 0.50(0.41,0.60) | 1845(1636,2063) | 0.54(0.47,0.61) | 0.33 (0.20 to 0.47) |  |
| Oceania | 15(12,18) | 0.70(0.57,0.85) | 40(32,52) | 0.80(0.65,0.98) | 0.50 (0.44 to 0.56) |  |
| South Asia | 1586(1035,1935) | 0.38(0.24,0.47) | 4130(3139,4905) | 0.35(0.27,0.42) | -0.43 (-0.52 to -0.35) |  |
| Southeast Asia | 1642(1418,1874) | 0.74(0.63,0.84) | 4107(3557,4596) | 0.78(0.67,0.87) | -0.05 (-0.17 to 0.06) |  |
| Southern Latin America | 326(301,359) | 0.80(0.73,0.88) | 803(704,861) | 0.94(0.83,1.01) | 0.66 (0.61 to 0.71) |  |
| Southern Sub-Saharan Africa | 151(125,170) | 0.65(0.54,0.74) | 401(362,433) | 0.86(0.77,0.93) | 1.23 (1.13 to 1.33) |  |
| Tropical Latin America | 801(753,839) | 1.06(0.97,1.12) | 2805(2424,3000) | 1.22(1.05,1.31) | 0.72 (0.65 to 0.79) |  |
| Western Europe | 3590(3263,3728) | 0.64(0.57,0.66) | 6563(5572,7043) | 0.60(0.52,0.64) | 0.07 (-0.08 to 0.22) |  |
| Western Sub-Saharan Africa | 205(169,240) | 0.27(0.22,0.32) | 612(471,732) | 0.38(0.29,0.44) | 1.33 (1.22 to 1.45) |  |

**Supplementary Table 3.** The number of DALYs rate and the age-standardized DALYs rate of NMSC in 1990 and 2019, and its temporal trends from 1990 to 2019. **Abbreviations:** NMSC, non-melanoma skin cancer; DALYs, disability-adjusted life years.

| Characteristics | 1990 | | 2019 | | 1990–2019 | P-value |
| --- | --- | --- | --- | --- | --- | --- |
|  | Number of DALYs cases(95% UI) | Age-standardized DALYs/100000 (95% UI) | Number of DALYs cases(95% UI) | Age-standardized DALYs/100000 (95% UI) | EAPC(95% CI) |  |
| **Overall** | 561854(518874,599141) | 14.44 (13.31, 15.42) | 1183233(1085365,1264545) | 14.67(13.45,15.67) | 0.20 (0.10 to 0.30) |  |
| **Sex** |  |  |  |  |  |  |
| Male | 346954(313033,371552) | 19.60(17.65,21.03) | 742528(681936,801690) | 20.05(18.44,21.64) | 0.20 (0.09 to 0.30) | <0.001 |
| Female | 214900 (196618,239155) | 10.29(9.42,11.40) | 440705(386735,485905) | 10.15(8.92,11.19) | 0.15 (0.04 to 0.26) |  |
| **Socio-demographic index** |  |  |  |  |  |  |
| Low SDI | 17356 (12145,20842) | 7.42(4.99,8.88) | 46988(32501,55894) | 8.91(6.00,10.62) | 0.70 (0.66 to 0.73) | <0.001 |
| Low-middle SDI | 66536(54893,76694) | 11.04(8.97,12.60) | 171320(152574,187362) | 12.73(11.23,13.96) | 0.68 (0.58 to 0.78) |  |
| Middle SDI | 151297(138962,163540) | 14.28(13.14,15.40) | 371884(335459,406888) | 15.39(13.80,16.80) | 0.55 (0.41 to 0.69) |  |
| High-middle SDI | 162720(153671,170572) | 15.85(14.96,16.61) | 279196(256518,299149) | 14.13(12.98,15.14) | -0.36 (-0.44 to -0.27) |  |
| High SDI | 163615(146298,184896) | 16.07(14.41,18.13) | 313081(269619,366999) | 16.71(14.40,19.57) | 0.21 (-0.09 to 0.50) |  |
| **Etiology** |  |  |  |  |  |  |
| basal-cell carcinoma | 562(257,1040) | 0.015(0.007,0.027) | 1703(782,3191) | 0.02(0.01,0.04) | 1.89 (1.46 to 2.33) | <0.001 |
| squamous-cell carcinoma | 561292(518444,598784) | 14.42(13.30,15.40) | 1181530(1084052,1262187) | 14.64(13.43,15.64) | 0.20 (0.10 to 0.30) |  |
| **Region** |  |  |  |  |  |  |
| Andean Latin America | 3158(2778,3682) | 17.12(15.10,19.19) | 10197(8174,12336) | 18.11(14.53,21.85) | 0.77 (0.63 to 0.91) | <0.001 |
| Australasia | 8225(7451,9252) | 37.47(33.81,42.10) | 19255 (16928,22065) | 38.70(34.12,44.17) | 0.09 (0.01 to 0.16) |  |
| Caribbean | 5426(5086,5802) | 22.34(20.90,23.63) | 13297(11188,15379) | 25.72(21.65,29.75) | 0.97 (0.85 to 1.09) |  |
| Central Asia | 4572(4018,4962) | 10.33(9.75,10.87) | 11296(9770,12536) | 15.58(13.32,17.17) | 2.32 (2.05 to 2.60) |  |
| Central Europe | 31617(28842,32562) | 20.73(19.12,21.42) | 35251(31182,39638) | 17.03(15.03,19.18) | -1.35 (-1.50 to -1.20) |  |
| Central Latin America | 21078(19859,21796) | 23.28(21.67,24.04) | 50602(43620,58376) | 21.48(18.45,24.77) | -0.53 (-0.58 to -0.49) |  |
| Central Sub-Saharan Africa | 1876(1281,2371) | 8.63(5.69,10.67) | 5712 (3940,7500) | 10.62(6.99,13.89) | 0.86 (0.77 to 0.94) |  |
| East Asia | 134005(117586,151286) | 14.64(13.50,16.19) | 332972(281033,384612) | 16.60(14.11,19.12) | 1.03 (0.76 to 1.30) |  |
| Eastern Europe | 40528(37541,42837) | 19.62(18.63,20.13) | 55392 (49181,62261) | 17.10(15.13,19.12) | 0.10 (-0.19 to 0.40) |  |
| Eastern Sub-Saharan Africa | 6419(3960,7956) | 9.27(5.38,11.61) | 18840(11109,23609) | 11.66(6.54,14.70) | 1.12 (1.06 to 1.18) |  |
| High-income Asia Pacific | 14511(13909,15006) | 6.75(6.28,7.02) | 26721(22957,28907) | 6.01(5.38,6.43) | -0.80 (-0.89 to -0.70) |  |
| High-income North America | 95064(79434,115631) | 20.64(18.21,23.74) | 195063(156597,245593) | 31.10(25.18,39.01) | 0.44 (-0.01 to 0.89) |  |
| North Africa and Middle East | 15491(13530,17898) | 9.01(7.84,10.10) | 38544(34653,43214) | 9.09(8.11,10.14) | 0.01 (-0.04 to 0.06) |  |
| Oceania | 390(317,481) | 13.82(11.33,17.28) | 1044(802,1387) | 14.56(11.53,18.65) | 0.41 (0.35 to 0.48) |  |
| South Asia | 38955 (26210,47451) | 6.99(4.98,8.26) | 90045(69417,107264) | 6.53(5.02,7.76) | -0.44 (-0.50 to -0.37) |  |
| Southeast Asia | 43611(37763,50247) | 17.10(14.94,18.91) | 97009(83976,109403) | 15.66(13.52,17.62) | -0.21 (-0.33 to -0.10) |  |
| Southern Latin America | 6634(6173,7217) | 15.44(14.48,16.17) | 13307(12046,14095) | 16.11(14.58,17.07) | 0.31 (0.27 to 0.34) |  |
| Southern Sub-Saharan Africa | 3367(2846,3757) | 13.56(12.72,14.51) | 8697(7845,9442) | 15.73(14.20,17.01) | 1.21 (1.09 to 1.32) |  |
| Tropical Latin America | 19475(18640,20297) | 21.33(20.28,22.08) | 53299(48209,56268) | 22.13(19.93,23.39) | 0.35 (0.28 to 0.42) |  |
| Western Europe | 62182(58498,64031) | 9.77(9.25,10.12) | 89704(80161,94929) | 9.92(9.08,10.46) | -0.24 (-0.38 to -0.11) |  |
| Western Sub-Saharan Africa | 5270(4411,6212) | 6.46(4.79,7.86) | 16983(12879,20756) | 7.72(5.90,9.26) | 1.30 (1.18 to 1.41) |  |

**Supplementary Table 4.** Global trends in the number of new cases, the number of deaths, the number of DALYs, the ASIR, the ASMR, and the age-standardized DALYs rate by sex from 2019 to 2044 predicted by the BAPC model. **Abbreviations:** ASIR, age-standardized incidence rate; ASMR, age-standardized mortality rate; DALYs, disability-adjusted life years; BAPC, Bayesian age-period-cohort.

|  | Number of new cases | | Number of deaths cases | | Number of DALYs cases | | ASIR/100000 | | ASMR/100000 | | Age-standardized DALYs/100000 | |
| --- | --- | --- | --- | --- | --- | --- | --- | --- | --- | --- | --- | --- |
|  | Male | Female | Male | Female | Male | Female | Male | Female | Male | Female | Male | Female |
| 2019 | 3682933 | 2670753 | 33244 | 22809 | 742528 | 440704 | 102.81 | 61.15 | 1.01 | 0.52 | 20.05 | 10.15 |
| 2020 | 3841597 | 2805309 | 33494 | 22933 | 749152 | 446139 | 96.67 | 58.92 | 0.85 | 0.42 | 18.77 | 9.46 |
| 2021 | 4023845 | 2913861 | 34580 | 23610 | 770552 | 454916 | 97.67 | 59.34 | 0.84 | 0.42 | 18.73 | 9.37 |
| 2022 | 4214902 | 3030135 | 35590 | 24245 | 791862 | 463340 | 98.67 | 59.79 | 0.83 | 0.41 | 18.69 | 9.28 |
| 2023 | 4413587 | 3154115 | 36458 | 24783 | 812588 | 471289 | 99.60 | 60.20 | 0.82 | 0.41 | 18.65 | 9.19 |
| 2024 | 4621315 | 3287602 | 37117 | 25167 | 832369 | 478575 | 100.43 | 60.56 | 0.82 | 0.40 | 18.59 | 9.10 |
| 2025 | 4845462 | 3436731 | 37550 | 25389 | 851463 | 485166 | 101.18 | 60.90 | 0.81 | 0.40 | 18.54 | 9.00 |
| 2026 | 5142099 | 3618554 | 38731 | 26103 | 876474 | 494307 | 101.92 | 61.23 | 0.80 | 0.39 | 18.48 | 8.90 |
| 2027 | 5475834 | 3826752 | 39845 | 26792 | 901872 | 503192 | 102.62 | 61.56 | 0.79 | 0.39 | 18.43 | 8.80 |
| 2028 | 5849642 | 4064343 | 40818 | 27396 | 927147 | 511724 | 103.22 | 61.84 | 0.78 | 0.38 | 18.38 | 8.70 |
| 2029 | 6271120 | 4337611 | 41582 | 27862 | 951956 | 519772 | 103.68 | 62.06 | 0.78 | 0.38 | 18.31 | 8.60 |
| 2030 | 6756268 | 4658728 | 42108 | 28176 | 976574 | 527251 | 104.05 | 62.25 | 0.77 | 0.37 | 18.25 | 8.50 |
| 2031 | 7392865 | 5052786 | 43456 | 29022 | 1008423 | 537437 | 104.39 | 62.42 | 0.76 | 0.37 | 18.18 | 8.39 |
| 2032 | 8148606 | 5522924 | 44750 | 29866 | 1041535 | 547557 | 104.67 | 62.58 | 0.75 | 0.36 | 18.12 | 8.29 |
| 2033 | 9045450 | 6084214 | 45899 | 30627 | 1075310 | 557387 | 104.83 | 62.68 | 0.74 | 0.36 | 18.06 | 8.18 |
| 2034 | 10117949 | 6760421 | 46822 | 31244 | 1109342 | 566697 | 104.85 | 62.72 | 0.73 | 0.35 | 17.99 | 8.07 |
| 2035 | 11423410 | 7589933 | 47498 | 31717 | 1144072 | 575438 | 104.79 | 62.73 | 0.72 | 0.35 | 17.92 | 7.96 |
| 2036 | 13168302 | 8638722 | 49055 | 32722 | 1188185 | 586861 | 104.70 | 62.74 | 0.72 | 0.34 | 17.85 | 7.85 |
| 2037 | 15367159 | 9950520 | 50553 | 33712 | 1234904 | 598101 | 104.57 | 62.73 | 0.71 | 0.34 | 17.79 | 7.74 |
| 2038 | 18161663 | 11606568 | 51910 | 34636 | 1283846 | 609111 | 104.34 | 62.68 | 0.70 | 0.33 | 17.73 | 7.63 |
| 2039 | 21753579 | 13723045 | 53019 | 35416 | 1334549 | 619584 | 104.00 | 62.58 | 0.69 | 0.33 | 17.66 | 7.51 |
| 2040 | 26459358 | 16478735 | 53841 | 36022 | 1387581 | 629330 | 103.60 | 62.47 | 0.68 | 0.32 | 17.59 | 7.40 |
| 2041 | 33149878 | 20194983 | 55728 | 37273 | 1454839 | 642578 | 103.22 | 62.36 | 0.67 | 0.32 | 17.53 | 7.28 |
| 2042 | 42367800 | 25200875 | 57573 | 38523 | 1527668 | 655682 | 102.83 | 62.26 | 0.67 | 0.31 | 17.48 | 7.17 |
| 2043 | 55263137 | 32046996 | 59231 | 39649 | 1605616 | 668323 | 102.39 | 62.13 | 0.66 | 0.31 | 17.42 | 7.05 |
| 2044 | 73642458 | 41587505 | 60575 | 40550 | 1688448 | 680152 | 101.87 | 61.97 | 0.65 | 0.30 | 17.37 | 6.94 |

**
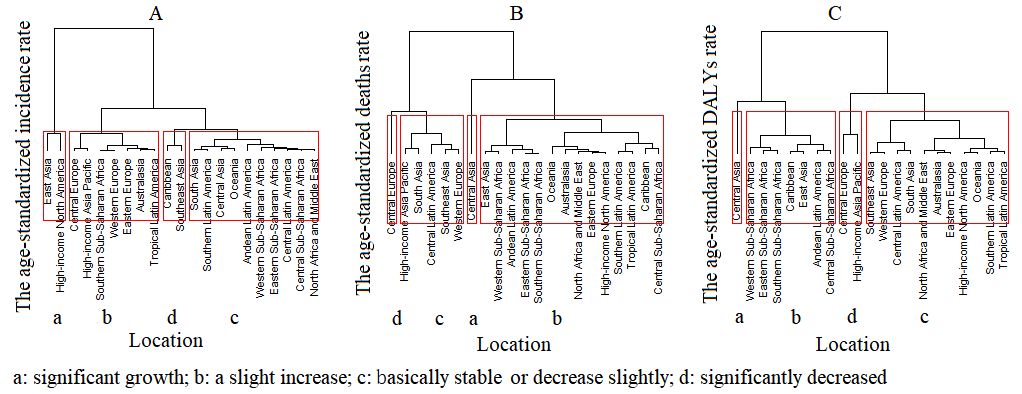
**

**Supplementary** **Figure 1.** Results of cluster analysis (a: significant growth; b: a slight increase; c: basically stable or decrease slightly; d: significantly decreased) based on the EAPC values of the ASIR **(A)**, the ASMR **(B)**, and the age-standard DALYs rate **(C)** from 1990 to 2019. **Abbreviations:** EAPC, estimated annual percentage change; ASIR, age-standardized incidence rate; ASMR, age-standardized mortality rate; DALY, disability-adjusted-life-year.


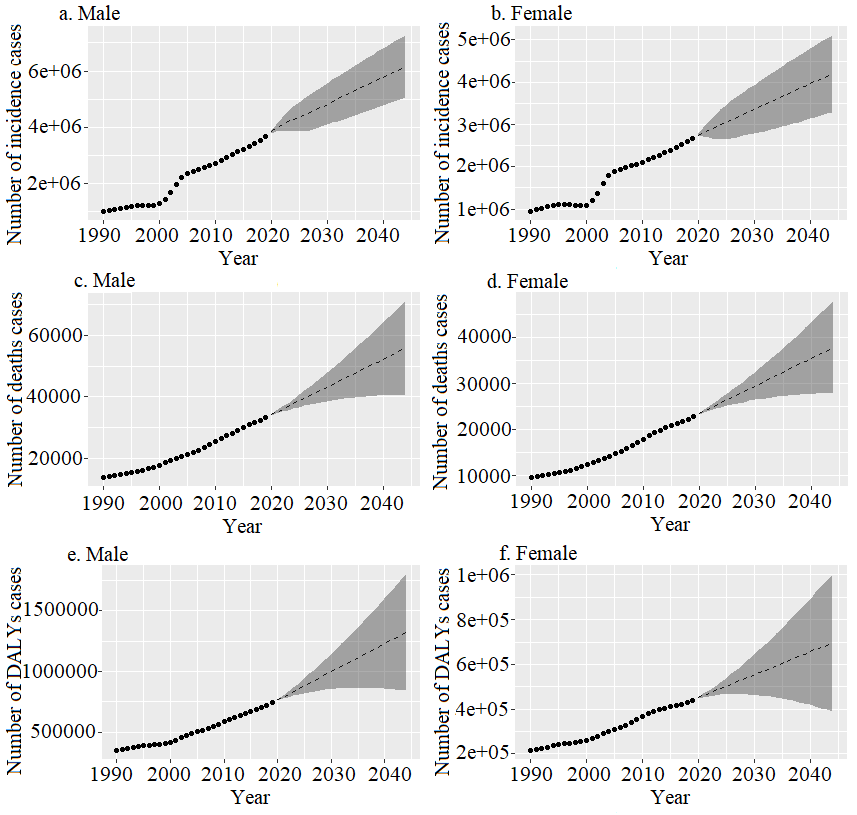


**Supplementary Figure 2.** Trends in the number of new cases **(a and b)**, the number of deaths **(c and d)**, and the number of DALYs **(e and f)** by genders globally: observed (before 2019) and predicted numbers of the ARIMA model (after 2019). Shading indicates the upper and lower limits of the 95% CIs. **Abbreviations:** DALYs, disability-adjusted-life-years; CIs, confidence intervals.


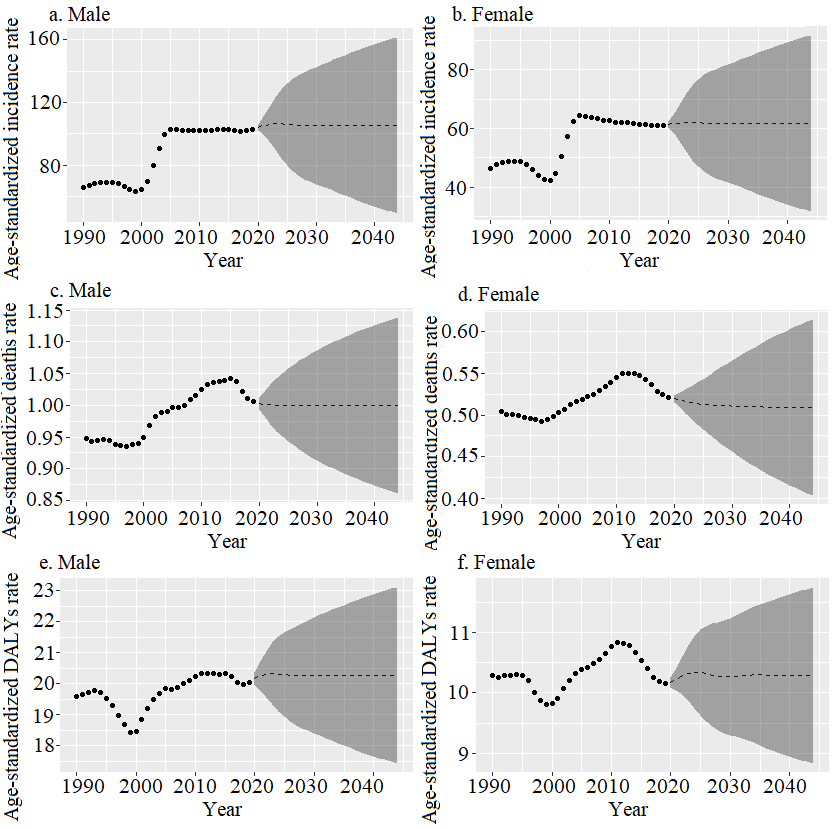


**Supplementary Figure 3.** Trends in the ASIR **(a and b)**, the ASMR **(c and d)**, and the age-standardized DALYs rate **(e and f)** by genders globally: observed (before 2019) and predicted rates of the ARIMA model (after 2019). Shading indicates the upper and lower limits of the 95% CIs. **Abbreviations:** ASIR, age-standardized incidence rate; ASMR, age-standardized mortality rate; DALYs, disability-adjusted-life-years; CIs, confidence intervals.
