# Supplementary figures and images for: Changing trends in the disease burden of non-melanoma skin cancer globally from 1990 to 2019 and its predicted level in 25 years
Source: BMC Cancer. 2022 Jul 30;22:836. doi: 10.1186/s12885-022-09940-3 (PMC9339183; doi:10.1186/s12885-022-09940-3)

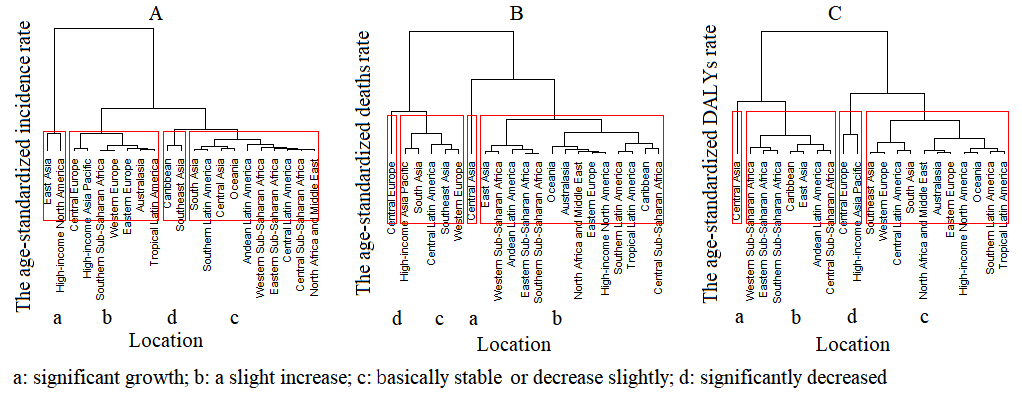

Supplement: Supplementary file 2 — Additional file 2 Supplementary Fig. 1. Results of cluster analysis (a: significant growth; b: a slight increase; c: basically stable or decrease slightly; d: significantly decreased) based on the EAPC values of the ASIR (A), the ASMR (B), and the age-standard DALYs rate (C) from 1990 to 2019. Abbreviations: EAPC, estimated annual percentage change; ASIR, age-standardized incidence rate; ASMR, age-standardized mortality rate; DALY, disability-adjusted-life-year. [file 12885_2022_9940_MOESM2_ESM.tiff]

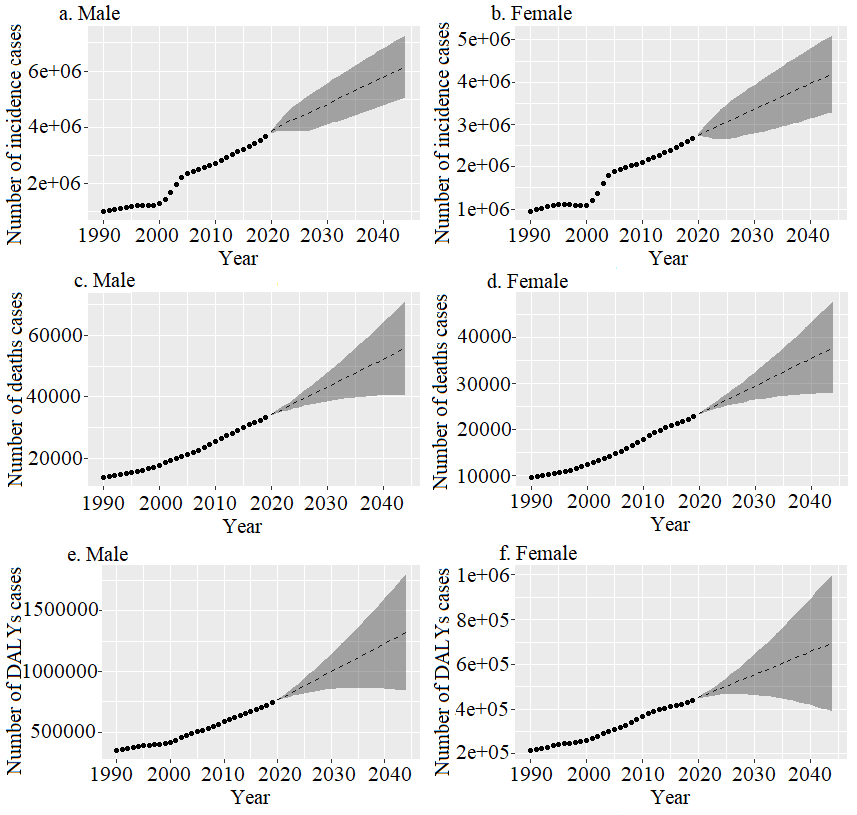

Supplement: Supplementary file 3 — Additional file 3 Supplementary Fig. 2. Trends in the number of new cases (a and b), the number of deaths (c and d), and the number of DALYs (e and f) by genders globally: observed (before 2019) and predicted numbers of the ARIMA model (after 2019). Shading indicates the upper and lower limits of the 95% CIs. Abbreviations: DALYs, disability-adjusted-life-years; CIs, confidence intervals. [file 12885_2022_9940_MOESM3_ESM.tiff]

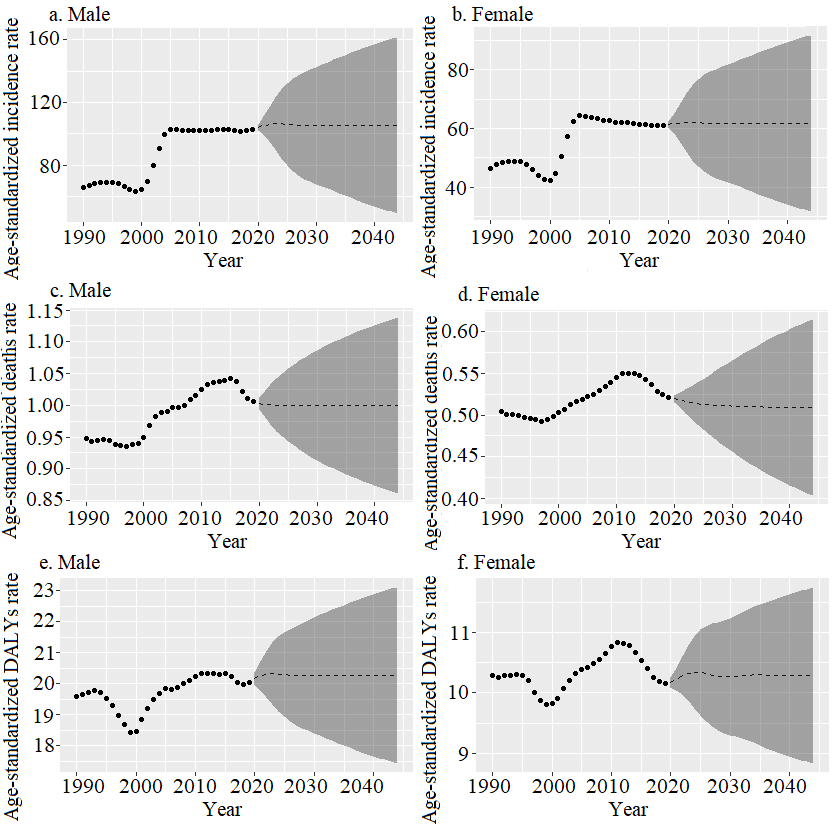

Supplement: Supplementary file 4 — Additional file 4 Supplementary Fig. 3. Trends in the ASIR (a and b), the ASMR (c and d), and the age-standardized DALYs rate (e and f) by genders globally: observed (before 2019) and predicted rates of the ARIMA model (after 2019). Shading indicates the upper and lower limits of the 95% CIs. Abbreviations: ASIR, age-standardized incidence rate; ASMR, age-standardized mortality rate; DALYs, disability-adjusted-life-years; CIs, confidence intervals. [file 12885_2022_9940_MOESM4_ESM.tiff]
